# Living tissues and dead objects in the eye: The first description of ocular immune privilege by J.C. van Dooremaal in Albrecht Von Graefe’s Archiv für Ophthalmologie in 1873

**DOI:** 10.1007/s00417-025-07077-0

**Published:** 2026-05-16

**Authors:** Martine J. Jager, Jacobus J. Bosch, Hanneke W. Mensink, Frédérique H. van Dooremaal, Frits C. Roest, Tero T. Kivelä, Claus Cursiefen

**Affiliations:** 1 Department of Ophthalmology, Leiden University Medical Center, PO Box 9600, Leiden, 2300 RC The Netherlands; 2 Department of Ophthalmology, University of Cologne, Cologne, Germany; 3 Eye Hospital, Rotterdam, The Netherlands; 4 Unitron Sonova, Vianen, The Netherlands; 5 Department of Ophthalmology, University of Helsinki and Helsinki University Hospital, Helsinki, Finland

**Keywords:** Ocular immune privilege, Rejection, Transplantation, Tolerance, Medawar, History of medicine

## Abstract

**Background:**

Immune privilege of the eye is considered to contribute significantly to the excellent survival of transplants in the anterior and posterior segment of the eye. While Sir Peter Medawar proved the existence of this phenomenon scientifically, a prior study by Dr. J.C. van Dooremaal already suggested the existence of ocular immune privilege and was published in this journal in 1873.

**Methods:**

The original 1873 German paper by J.C. van Dooremaal in Albrecht von Graefe’s Archiv für Ophthalmologie was translated into English and commented on by immunologist Dr. J. Wayne Streilein and other eye specialists. Family of Dr. van Dooremaal supplied information about the author himself.

**Results:**

Dr. van Dooremaal was a military doctor when he was confronted with a severe epidemic of trachoma while serving at the Government Institution of Benevolence in the Netherlands (now a UNESCO heritage site). While finding a solution to fight the epidemic, he became familiar with the eye clinic of professor F.C. Donders in Utrecht. He subsequently trained as an ophthalmologist, and worked on a thesis on implantation of foreign tissues in the anterior chamber of different animals. He observed that a piece of skin of a white mouse placed in the eye of a dog survived amazingly long and was integrated into the tissues of the anterior chamber instead of rejected. According to Dr. J. Wayne Streilein, this was the first experimental demonstration of ocular immune privilege.

**Conclusion:**

The 1873 paper by Dr. J.C. van Dooremaal can be regarded as the first description of ocular immune privilege, and as Dr. J. Wayne Streilein wrote in 2001: Dr. van Dooremaal is the FATHER of immune privilege – although he could not call it that since immunology had not yet been discovered.

**Supplementary Information:**

The online version contains supplementary material available at https://doi.org/10.1007/s00417-025-07077-0.

## Introduction

Immune privilege is considered a condition in which normal immune responses are not functioning: when an antigen is introduced in an immune-privileged site, it either does not induce an immune response or the induced response is locally inhibited and is not as effective as elsewhere in the body. A typical example is the unusually good survival of non-matched corneal grafts and especially lamellar grafts (Hori et al. 2007, Van Essen et al. 2015, Hos et al. 2019).

Aside from the eye, typical immune-privileged sites are the brain and the testis. In 1948, Medawar described prolonged survival of heterologous tissue grafts placed into the anterior chamber of rabbits and coined the term “immune privilege” to describe this phenomenon (Medawar 1948). However, we now know that more than 75 years earlier, the phenomenon had already been observed and published by Dr. Jacobus Christiaan van Dooremaal, a student of Professor Franciscus Cornelis Donders in Utrecht, The Netherlands.

The paper was published in 1873 in Albrecht von Graefe’s Archiv für Ophthalmologie in Berlin under the title “Die Entwickelung der in fremden Grund versetzten lebenden Gewebe” van Dooremaal (1873). This paper describes the experimental work that van Dooremaal performed for his PhD thesis, which was published in Dutch and which he defended on June 3, 1873, in Utrecht, The Netherlands.

The first author became aware of the work of van Dooremaal when she accidentally came across his Dutch thesis when the librarians wanted to throw away a book that listed the ophthalmology books available at the Leiden University Library. When leafing through, the first author saw the title of one of the books: “Levende weefsels en doode voorwerpen in het oog” (“Living tissues and dead objects in the eye”) and requested the book through the librarian. She discovered that the book was a thesis from one of the PhD students of Donders and described a series of experiments that had been performed in dogs and rabbits. Subsequent research showed that the thesis had been published as a paper in German.

## Albrecht Von Graefes Archiv für Ophthalmologie in 1873

The paper “Die Entwickelung der in fremden Grund versetzten lebenden Gewebe” (“The development of living tissues placed in strange soil”) describes a series of experiments where tissues or material was placed into the anterior chamber of different experimental animals:

Experiment I. Moderately large dog. Piece of rolled-up paper.

Clinical: Corneal inflammation, staphylomatous expansion.

Pathology: Cystic foreign body between the iris and the cornea with thick epithelium.

Experiment II. White rabbit. Piece of the middle part of a hair.

Clinical: Hardly any response.

Pathology: Small extension with vessels coming from the iris.

Experiment III. Small dog. Piece of bulbar conjunctiva.

Clinical: Slight exudative response.

Pathology: Conjunctival tissue is fused with the iris and the cornea.

Experiment IV. Large dog. Piece of dorsal skin of a newborn white mouse.

Clinical: A relatively severe inflammation subsided quickly; piece remained in place.

Pathology: skin tissue is fused with the cornea and with the thickened iris.

Experiment V. Dog. Piece of skin from the inner surface of the animal’s ear.

Clinical: Extrusion of the skin piece, with massive iritis.

Pathology: Iris is attached to the cornea.

Experiment VI. Large gray rabbit. Piece of mucosa from the inner surface of the lip.

Clinical: Mild inflammatory symptoms. Small homogenous tumor on the iris.

Pathology: Implanted mucosa has adhered to the iris and the cornea.

## Implications

The most important experiment is number IV, where a xenograft of mouse skin was implanted into the eye of a large dog, and remained in situ. Similar experiments are nr III and nr VI; however, in these cases, the implant was most likely autologous tissue that was placed in the eye and remained in situ.

As the first author had previously worked in the laboratory of Dr J. Wayne Streilein, the world expert in ocular immune privilege, she reported her discovery to him in 2001 by e-mail. On October 3, 2001, he responded enthusiastically: “Van Dooremaal is the FATHER of immune privilege – although he couldn’t call it that since immunology had not yet been discovered”. He wrote further: “Both Jerry Niederkorn and I have almost always included a reference to van Dooremaal in our reviews of ocular immune privilege (he then references the 1873 Graefe’s Archiv paper). I have a much-trampled Xerox copy of this article which I could copy and send to you if you cannot find a copy in Leiden. What fun!!!!!”

In his paper “Immunoregulatory mechanisms of the eye”, Progress Retinal Eye Res, 1999; vol 18, 357–370, https://doi.org/10.1016/S1350-9462(98)00022-6”, Streilein refers to van Dooremaal’s work:

“Dooremal’s observation that human tumor fragments would grow in the anterior chamber of rabbit eyes (but not elsewhere) was the first evidence that the anterior chamber was immune privileged, and Medawar’s experiments formally described immune privilege as a feature of the anterior chamber. Maumenee capitalized on Medawar’s discoveries and explanations and demonstrated that the extraordinary success of orthotopic corneal transplants was due to the privileged nature of the cornea (Maumenee 1951).“.

Streilein had most likely not seen the full paper, as he referred to implanting tumor fragments in the eye, not tissues. This is probably related to a misunderstanding, related to one of his earlier papers (Streilein et al. 1980), in which he mentions the work of Greene (1938, Medawar 1948). When a proper English translation had been prepared, it was sent to Streilein in 2002. Streilein responded: “Van Dooremaal’s mouse skin experiment demonstrates the existence of immune privilege (viewed from our retrospective perspective) – but he never injected “tumors” into the eye. Greene appears to be the guy that did that)”. It was indeed H.S.N. Greene who performed extensive studies on the transplantation of tumors and endocrine tissues in the anterior chamber of the eye and implied that this site might enjoy a degree of immunological privilege (Greene 1938, Medawar 1948).

## Immune privilege of the eye

It is now known that placement of tissues in the anterior chamber leads to the induction of systemic regulator T cells that may lead to survival of corneal transplants and tumor cells in the eye that would otherwise be rejected. This phenomenon of ocular immune privilege is the product of a range of passive and active processes: a lack of lymph vessels, a low expression of major histocompatibility molecules on many ocular tissues, the presence of immunosuppressive molecules in the ocular fluids and intraocular cells, and a specific active immunosuppressive mechanism. In the 1970’s and 1980’s, the mechanism of this so-called anterior chamber-associated immune deviation (ACAID) was elucidated by the laboratories of Streilein and Niederkorn (Streilein 1980, 1985; Niederkorn 2019; Clahsen et al. 2023). Antigens in the anterior chamber are picked up by macrophages that have been incubated in TGFbeta-containing anterior chamber fluid, and transported through the bloodstream to the spleen (Streilein 1981). Interactions between the macrophage and T cells in the spleen lead to the development of regulator T cells, that for instance are capable of suppressing the development of delayed type hypersensitivity reactions (Niederkorn 2019 Proctor).

We assume that Streilein knew of van Dooremaal’s paper through his supervisor, Dr Rupert E. Billingham. Streilein started his academic career at the University of Pennsylvania School of Medicine as associate professor at the Department of Medical Genetics under the leadership of Billingham, and then accompanied Billingham when he moved to the University of Texas Southwestern Medical School in Dallas. “Bill” Billingham had himself trained at University College London under the mentorship of Sir Peter Medawar and co-authored one of the most important paper’s that introduced immune privilege (Billingham et al. 1954). Streilein et al. (1980) refers to a paper by Barker and Billingham (1978), which refers to van Dooremaal’s paper. However, Streilein mentions that the first experimental work was done in the 1940s. referring to Green’s 1949 paper and Medawar’s. Medawar and Sir F. Macfarlane Burnet shared the 1960 Nobel Prize in Physiology or Medicine “for discovery of acquired immunological tolerance.” We have not found any references to the work of van Dooremaal in Medawar’s papers or Billingham’s papers before 1978.

Still, van Dooremaal’s work was regularly cited early in the 20th century (Von Tiesenhausen 1909; Hegner 1913; Keysser 1913; Beyer 1937, Lucké 1939) and was known to transplantation immunologists in the 1950’s: the paper in Graefe’s Archiv is mentioned by Sir Michael Woodruff (1950, 1960): “As regards the anterior chamber, successful heterotransplantation of tumour and embryonic tissue, and successful homotransplantation of tumour tissue, embryonic tissue and certain normal adult tissues have been reported by various workers. The use of this site was first described by Van Dooremaal (1873)”.

At the time of correspondence with Streilein in 2001 and 2002, we could not find personal data about Van Dooremaal, the author of the thesis and the paper, and did not publish our findings. This changed when the first author was approached by Frédérique van Dooremaal, granddaughter of J.C. van Dooremaal, who had collected information and saw the first author’s professor’s oration at Leiden University on the internet, in which van Dooremaal’s thesis was mentioned (Jager 2016). As the original text is of great historical value, we decided to combine a full English translation of the German paper (online) with the personal history of the author.

## Van Dooremaal’s life

**Fig. 1:**
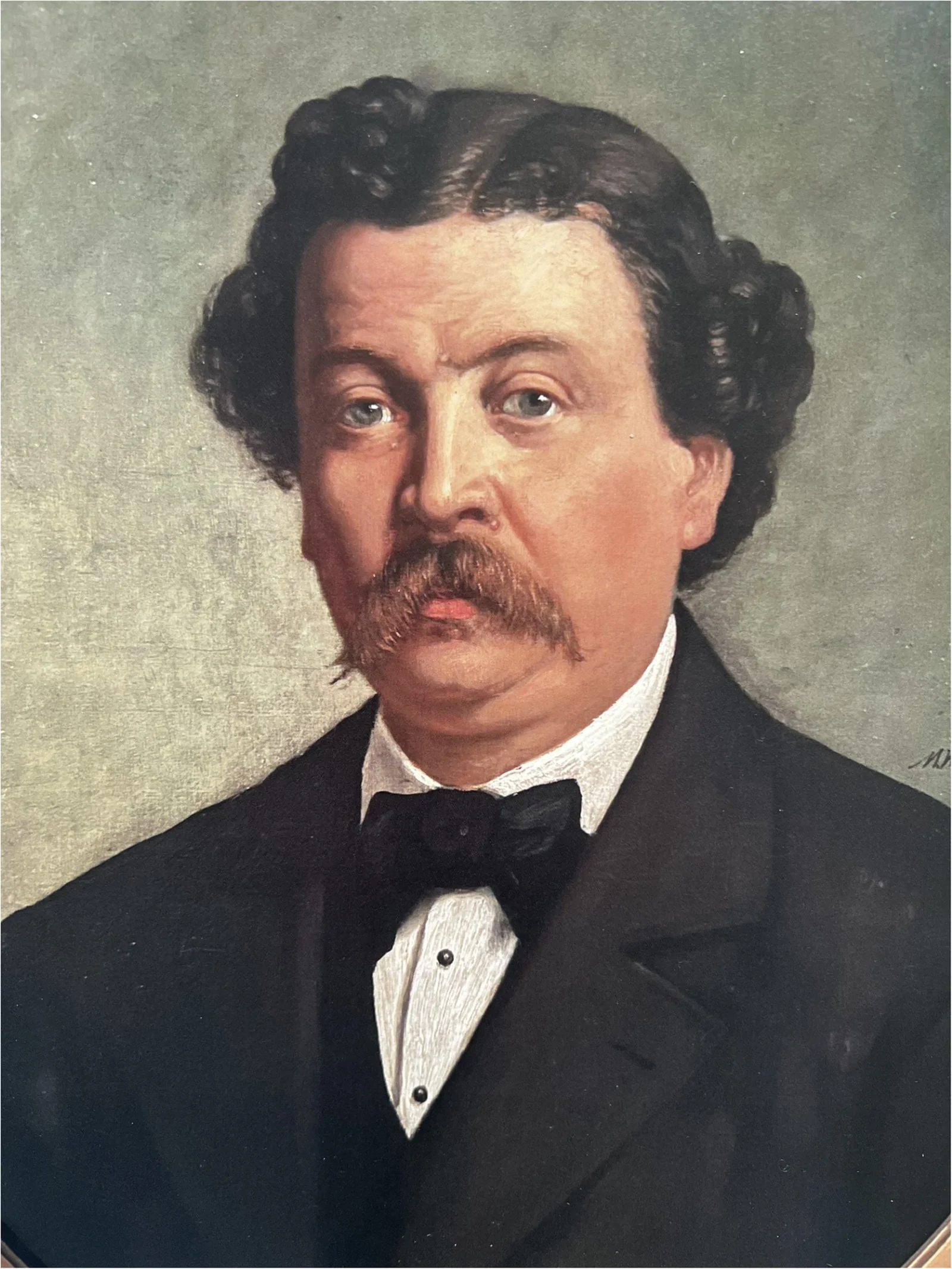
Portret of Dr J.C. van Dooremaal, painted by Martinus Wilhelmus Liernur (1833–1901) in 1879

Jacob Christiaan van Dooremaal was born in 1837 in a local skipper’s family in Breda, The Netherlands, and started his education to become a Health Officer at the School for Military Doctors at Sea in 1854. As Medical Officer, he joined a variety of steamships, travelling to the East Coast of the United States of America to protect the Dutch merchantmen during the American Civil War (1861). In 1864, he was ordered to the Dutch East Indies, returning in 1868, when he got married in The Netherlands. (Fig. 1)

Van Dooremaal then joins the Government Institution of Benevolence Ommeren/Veenhuizen in 1868 as Geneesheer (local physician). The so-called Colonies of Benevolence had been founded in 1818 to fight poverty. In 2021, the Colonies of Benevolence were declared a UNESCO World Heritage Site. The Institution offered pieces of land and houses to people who were less well off. Educating poor people from the big cities in agricultural skills would offer them a better life so that they would be able to provide for themselves. In addition, housing was built for orphans, beggars, and layabouts. These places were called “Colonies” because they were built in the middle of nowhere. Specifically, no shops were available that might sell liquor. However, many people were placed together, allowing for the spread of infectious diseases.

Van Dooremaal was confronted with many patients with either trachoma characterized by a chronic course or with ophthalmia granulosa (granulomatous eye disease) with a purulent conjunctivitis, followed by keratitis. These diseases had been affecting people in the Colonies for years and as beggars and orphans shared the same building, many children became infected. He asks for advice in Utrecht and decides to become an ophthalmologist. He registers as a student at the University of Utrecht in 1869 (Utrecht Student Almanak). Working with professor H. Snellen, they come up with one important solution: the separation of the beggars and orphans by housing them in different buildings. After the death of his wife in 1870, he decides to stay on at the Institution for another 2 years. In 1872, van Dooremaal calls attention in the national newspaper to the problem of eye diseases in the government institutions: while he managed to reduce the number of infected people by placing them in a separate clinic and treating them, he now calls upon the government not to close the eye clinic, as the disease will then spread again (Algemeen Handelsblad 17 September 1872).

Between 1872 and 1873, van Dooremaal joins Professor F.C. Donders in Utrecht and works on his thesis. We assume that he also learned about clinical care during these years. He subsequently settles as an ophthalmologist in The Hague in 1873. He becomes a distinguished specialist, Editor-in-Chief of the Nederlands Tijdschrift voor Geneeskunde, and continues his interest in infectious diseases and their prevention, especially cowpox vaccinations. He meets Queen Sophie of Würtenberg, the wife of King Willem III of The Netherlands in a meeting about the importance of such vaccinations (Het Vaderland, 23 December 1874), as well as King Leopold of Belgium (Algemeen Handelsblad, 25 September 1892).

Aside from running his own practice, van Dooremaal attends to non-paying patients and becomes a physician for the prisoners at the Scheveningen prison. His work includes treating children with scrophulosis, the most common condition that led to admission of children to the Sophia Foundation in Scheveningen, a bathing facility for the poor. He becomes involved in several more foundations for children and blind people, joins the Board of the Nederlandse Maatschappij tot Bevordering der Geneeskunst (The Dutch Society for Improving Medicine), helps with the organisation of the festivities for the 70th birthday of his majesty the King in 1887 as well as of Professor F.C. Donders in the same year.

On June 20, 1893, van Dooremaal dies from pneumonia at the age of 55 years. His funeral is attended by many friends and military colleagues, as well as by speakers from the organisations to which he belonged and grateful patients, who threw flowers on his grave.

## The thesis of Van Dooremaal

We can learn more about the background for his experiments from the introduction of his thesis. J.C. van Dooremaal defended his thesis in Utrecht, The Netherlands, on June 3, 1873. In the Preface of his thesis, he writes: “After leaving my office as an Ophthalmologist at the Government Institution Ommerschans and Veenhuizen, which I had occupied for four years, I wished, prior to establishing myself as an ophthalmologist in The Hague, to acquire the title of *medicinae doctor* at the Academy of Utrecht. “.

During the winter of 1872–1873, he had the privilege of participating in the ophthalmologic colloquium, which was held by Professor Donders every Wednesday evening and attended by those interested in ophthalmology. One evening, van Dooremaal reviewed a paper by Monoyer, Professor at Nancy, France, entitled *Epithélioma perlé ou margaritoïde de l’iris* (1872). In this article, Monoyer describes an eye in which, after an injury, a tumor of mother of pearl appearance developed on the iris. After extirpation, the tumor was shown to be made up of squamous cells, compatible with the cells of keratinized epidermis, apart from some grainy dust and cholestearine crystals. In addition, the iris showed a second smaller tumor, which looked like a pearl, and through which a hair extended. This hair, without a hair bulb, had surely detached during the accident and had been implanted in the eye from outside.

A discussion ensued about different types of iris cysts. Rothmund (1871, 1872) differentiated two types of cysts: epidermoid, which were made up of epidermal cells, and cysts with an aqueous content lined by epithelium. According to Rothmund, both types were the result of injury, to which Monoyer added that while epidermoid cysts were of a traumatic nature and resulted from implantation of skin or hair bulbs, other cysts could possibly represent proliferation of loosened, implanted epithelial cells from the conjunctival border of the cornea, or they resulted from closure of a part of the iris, e.g. by posterior synechiae. It was on this basis that Donders proposed to perform a study regarding the phenomenon of cyst formation: one could test this by placing foreign bodies, especially living tissues, in the anterior chamber in contact with the iris, and then later study the eyes anatomically.

Van Dooremaal set out to implant dead foreign bodies, such as paper, cork, shots of hail, and hairs, as well as living tissues, such as palpebral and bulbar conjunctiva, mucosa of the lips, skin of several animals, periosteum of a cat, and human epidermis. He used twelve rabbits and three dogs as recipients (see description of experiments above). The implanted pieces were placed on the iris with a fine needle, far enough from the wound by pushing on the cornea. The results were published in his thesis in Utrecht (“Over de gevolgen van het invoeren van levende weefsels and doode voorwerpen in het Oog”), and in von Graefe’s Archiv für Ophthalmologie: “Die Entwickelung der in fremden Grund versetzten lebenden Gewebe.”

As mentioned, after the first author informed Dr Streilein about her findings, he called attention to mentioning van Dooremaal’s German text in a paper by his wife and him (Stein-Streilein 2002) and by Niederkorn (2019), where they describe the proper experimental findings, although they do not know the background of the experiments, which was not mentioned in the German paper: “The roots of immune privilege reach back two centuries to an observation made by the Dutch ophthalmologist Van Dooremaal (1873). In an attempt to identify the etiology of cataracts, van Dooremaal inserted a variety of foreign objects and tissues into the eyes of rabbits and dogs. Although he failed to discover the cause of cataracts, he observed a significant prolongation in the survival of mouse skin grafts placed into the anterior chamber of the dog eye. Another 75 years would pass before Medawar ‘rediscovered’ the prolonged survival of foreign tissue grafts placed into the anterior chamber of rabbits and coined the term immune privilege to describe this phenomenon (Medawar 1948).“.

Conclusion: van Dooremaal’s thesis and his paper “Die Entwickelung der in fremden Grund versetzten lebenden Gewebe” in Albrecht von Graefe’s Archiv für Ophthalmologie is the first description of the phenomenon of immune privilege of the eye and, therefore, a very important publication by laying the ground to modern transplant immunology and the ocular immune privilege.

## Supplementary Information

Below is the link to the electronic supplementary material.

Supplementary Material 1 (DOCX 36.1 KB)

## Declaration

### Competing intrests

There are no competing intrests.
