# Supplementary material for: Living tissues and dead objects in the eye: The first description of ocular immune privilege by J.C. van Dooremaal in Albrecht Von Graefe’s Archiv für Ophthalmologie in 1873
Source: Graefes Arch Clin Exp Ophthalmol. 2026 May 16;264(9):2661–7. doi: 10.1007/s00417-025-07077-0 (PMC13578108; doi:10.1007/s00417-025-07077-0)
Supplement: Supplementary file 1 [file 417_2025_7077_MOESM1_ESM.docx]

Die Entwickelung der in fremden Grund versetzten lebenden Gewebe, or:

The development of living tissues transplanted into foreign soil.

by Dr. J.C. van Dooremaal.

See Plate IV.

*) R o t h m u n d, zur Pathogenese der Iriscysten. Sitz.-Ber. Der ophth. Ges. 1871. Monatsbl. f. Augenh. IX. S. 397.
D e r s e l b e. Ueber Cysten der Regenbogenhaut. Monatsbl. f. Augenh. X. S. 189-223.
M o n o y e r. Epithé1ioma perlé ou margaritoïde de l'iris. Gaz. méd. de Strassbourg. Juni 1. 1872. u. Ann. d'Ocul LXVII. 249-259.
Also compare:
S t ö b e r. Gaz. hebd. I. 2. p. 55. 1855.

v. G r a e f e. Arch. f. Ophth. III. 2. p. 412. VII. 2. p. 139.

B o w m a n. Lect. On the parts etc. p. 75.
H u l k e. Ophth. Hosp. Rep. VI.

K n a p p. Arch. f. Augen- u. Ohrenheilk. I. 2, p. 175.

A r l t, Krankh. d. Auges II. Bd. S. 109.
S c h w e i g g e r. Sitz.- Ber. d. ophth. Ges. 1871. Monatsbl. f. Augenh. IX. S. 405 –

And others

When the eye undergoes a penetrating injury, it is not uncommon for either true cysts to develop on the iris, or epitheliomas or epidermoidomas, caused by the penetration of hairs, skin particles or mucous membranes from the eyelids that were injured at the same time. *

In order to gain more insight into the origin and development of these processes, the effect of various substances and living tissues introduced into the anterior chamber of the eye of dogs and rabbits was investigated at the instigation and under the direction of Professor Donders. Here I would like to restrict myself to a brief overview of the results obtained, preceded by a description of some of the most important experiments (which are reported in more detail elsewhere).

The following were introduced: foreign dead bodies, such as paper, cork and shot grains, then hair, and as living tissue, conjunctiva palpebrarum et bulbi, mucous membrane from the lip, the skin of various animals (including newborn white mice), the periosteum of the cat and the human epidermis.

With a standard iridectomy lance, a wound was made on the edge of the cornea near the sclera in a perpendicular direction to the axis, large enough to insert a usually very thin part of 1 mm square. While the wound was being made, the watery fluid had usually already flowed out. The introduced body was placed onto the iris with a fine needle, and it was usually possible to move it to a sufficient distance from the wound on the iris by rubbing the cornea. The signs were observed for 1-4 months (a longer period was not available to us). The animals were then killed. The removed eyes were placed in Müller 's fixative for 3-6 weeks and then in 25° alcohol for 3 to 4 days.

Experiment I. Moderately l a r g e d o g. On January 18th, a small piece of rolled up white paper was introduced in the usual manner and pushed down onto the iris.

Inflammation of the conjunctiva occurs, with strong injection of the vessels and mucus secretion, soon followed by an inflammation of the cornea opposite the foreign body, where after seven days, a noticeable staphylomatous expansion is already visible. While the conjunctivitis subsides, the keratitis continues and the staphyloma increases, but in a localized area. On February 4, the eye was removed.

The cross-section shows that the foreign body, Plate IV, Fig. 1 P. (not shown in the drawing), surrounded by a layer of newly formed tissue (4 and 4'), is incorporated into the locally thickened iris ii, with its front fused with the curved cornea. Opposite the middle, where the cornea is very thin, Descemet’s membrane (d) is lacking, which otherwise looks normal.

At the place of the adhesion, the pigment of the iris stroma J continues more or less, so that the cystic foreign body is actually enclosed in the thickened tissue of the iris. The encysting tissue is very notable, as can be seen under high magnification (cf. Fig. 2, corresponding to the * in Fig. 1).

We see on the cornea (C) Descemet’s membrane, internally integrated to the tissue of the thickened iris, in which we still find pigment (3'); next comes the fibrous tissue (3) of the cyst. On the inner surface of this tissue is a very thick layer squamous epithelium (compare 4 and 4', both in Fig. I and II). The cells of the outermost layer 4' have a cylindrical shape, followed by small, round cells and larger and larger, increasingly flattened ones. All cells, even the innermost ones, have distinct nuclei. The posterior side contains no less than 30 layers of these epithelial cells. In shape, the epithelium differs little from that on the anterior surface of the cornea; however, it is several times thicker and the cells are larger.

In general, the disease process of the experiment reported here requires no explanation. However, two points require our attention. First, the extension of the cyst surrounding the foreign body into the iris tissue: apparently, the iris adhered to the developing cyst from the beginning and the anterior layers of the iris were pulled forward by the developing cyst, so that the cyst became incorporated into the iris tissue.

Secondly, the layered epithelium covers the inner surface of the cyst, which is otherwise very thin. Did it develop completely independently, or did it develop out of iris epithelium, or were cells from the corneal epithelium introduced into it? I do not dare to decide. The fibrous layer of the cyst was probably much thicker at the beginning, but expanded and thinned as the thick layers of the epithelium developed.

III. Experiment. W h i t e R a b b i t. A piece cut from the middle of a hair is placed on the upper outer quadrant of the iris, so that it rests entirely on the iris without extending over the pupil.

The wound heals quickly; initially, there are slight signs of inflammation, which soon disappear completely. At the inner end of the hair particle, a small protrusion develops from the iris, which progresses slowly under the hair, leading to a 1 mm-sized lesion.

On February 6, when the eye was removed, the tumor was approaching as far as the middle of the hair. The hair has remained unchanged and is not connected to the enlarging mass.

The tumor consists of large, branching, more or less developed papillae, originating from the thickened iris (Fig. 9), the vessels of which (v) are extremely swollen. In the middle of the papillae and their extensions are some quite large blood vessels (v'), which are located in a layer of partly homogeneous, partly fibrous connective tissue (f).

Incidentally, the papillae are covered with normal squamous epithelium, in most places consisting only of a single layer of cells, in others, it seems, of many.

In the large papillae, one finds numerous groups of colored blood corpuscles b. Some of these are evidently located in the vessels: whether they also occur outside the vessels is less easy to determine; however, it is certain that they do not occur in the epithelium.

We cannot explain the origin of this peculiar proliferation. It is closely connected with the iris tissue. Compared to the papilloma, its vessels are greatly swollen and produce large branches in the axis of the papillae and all their branches, whose fibrous tissue is also directly
connected with the stroma of the iris. To all appearances, the proliferation originates from the iris, although in its normal state it has neither papillae nor villi on its front surface. It is hardly conceivable that the hair or substances accidentally introduced with the hair have a specific influence. The only thing that can be done to clarify this is to repeat the experiment of introducing a hair.

III. Experiment. S m a l l d o g. On February 3rd, a piece of the bulbar conjunctiva was placed onto the iris in the usual way. At first, inflammatory symptoms appeared with the formation of exudate on the inserted tissue. When the inflammation subsides, the tissue gradually reappears and looks to be fused to the cornea in front and to the pupillary margin in the back, while the pupil retained its round shape and the inner eye was healthy.

On March 17^th^, the eye was enucleated.

It turns out that the inserted piece of the conjunctiva, as was well recognized during life, is fused to the cornea and the iris, less firmly to the iris, but very intimately to the cornea.

In most areas, Descemet’s membrane (d, Fig. 4) can be identified clearly, indicating that fusion of the conjunctival tissue and the membrane occurred. In some sections, however, Descemet’s membrane had lost its sharp outlines and its characteristic strong light refraction, and showed some curved lines.

The piece itself consists of fibers that radiate from one point (e), are colored deeply carmine and have the shape of large spindle-shaped cells. On the surface (2), one can see partly iris tissue and partly newly-formed connective tissue. Pigment cells from the stroma of the iris can also be seen on the posterior surface (2').

Granular pigment has penetrated deeper in 3, apparently into the implanted tissue, and in some sections even traces of pigment are visible in the cornea. Sporadically, cell proliferations do occur, but nowhere can the original epithelium of the implanted tissue be recognized.

It turns out that the implanted living tissue, after its connection with the iris and cornea and after alterations in its structure, lives on, which can be inferred from the connection of the vessels visible here and there on the surface.

IV. Experiment. L a r g e d o g. On January 18th, a piece of the dorsal skin of a newborn white mouse was placed with its wound side onto the iris, whereby the fine white hairs extended freely into the anterior chamber. The relatively severe inflammation soon subsided. Soon, the piece was surrounded by an exudate and remained in place.

Gradually, the implanted piece of skin seemed to increase in size and approached the cornea, which, on contact, showed severe signs of inflammation and became cloudy. The iris tissue on which the piece rested, was lifted up at one spot so that it came forward. On March 17, the globe was removed.

There was no external inflammation. The implanted tissue, which has noticeably increased in size, is attached to the cornea anteriorly and to the iris posteriorly and has lifted the iris up so that the iris fibers on the opposite side are located deeper. The media are transparent. From the sections it can be seen that the introduced piece of skin (H. Fig. 5) has shrunk almost to a ball, lying between the cornea (C) and the iris (I), intimately fused with both. The corneal epithelium (e) continues in a regular fashion, also opposite the piece of skin, only to appear less regularly on the outside and on the sclera (S).

The actual corneal tissue (C) shows little change, is thickened at (1) and is noticeably thinner at (2) opposite the frontal curve of the skin tissue.

Descemet’s Membrane (d) seems to come forward at 3 although it may also dissolve into a more loose tissue. However, curved sections of the Descemet’s Membrane, twisted due to shrinkage, appear again at the borders at (4).

The skin fragment (H) is yellowish in color, shows multiple sections of hairs, and some tips seem to penetrate into the cornea and the iris, but is otherwise a fairly homogeneous tissue in which well-developed morphological elements are not visible.

At (3), there is a small layer of newly-formed tissue that seems to extend from the cornea, and is crimson-colored. At the border between the cornea and the skin (at 5 and 6), the tissue merges into the corneal tissue without a sharp demarcation. Posteriorly, it is surrounded by the thickened iris tissue and is intimately fused with it, while around it lie spherical groups of pigment granules (b), which evidently come from the iris, which is poor in pigment due to new interstitial tissue (a).

Near the posterior surface, pigment is present in all its glory up to the pupillary border (p).

V. Experiment. D o g. On December 20th, a piece of skin taken from the inner surface of the animal's ear was placed on the iris.

A rather large iris prolapse occurred, making it impossible to move the piece of skin extruded from the wound. Subsequently, a severe iritis developed and the piece of skin that had been introduced came out through the wound. The iris was displaced towards the wound, closing the pupil almost completely; exudate was seen in the part that was still open.

On March 17, the eye was removed. Under the microscope, we notice that the tissue had become very flaccid and that iris tissue shows little pigment (J. Fig. 6). The implanted tissue had moved into the corneal tissue for up to half the thickness of the cornea (C.). Here, newly-formed tissue that contains no pigment and is carmine colored is visible in the iris tissue. At the periphery of the place where the iris is inserted in the cornea, Descemet’s Membrane (d) suddenly stops and appears to split into fine bundles, which extend like a fan towards the central area. Where the iris is attached to the cornea, pigment (p) has spread both centrally as well as peripherally in a linear pattern, which follows the course of the corneal lamellae.

In most areas, this pigment does not extend beyond half the corneal thickness; only in one location (scar 1 and 1 ^1^ ) it extends obliquely to very close to the anterior surface of the cornea. By the way, the corneal tissue appears to be normal. The epithelium of the anterior surface of the cornea is completely intact. Opposite the place where the iris is located in the corneal tissue, the epithelium contains pigment granules. Granules are almost directly touching the epithelium. Under high magnification we notice that the lamellae of the corneal tissue have a curved course towards the pigment line.

Particularly worthy of our attention here are the scars l and 1' of the wound, through which the small piece of skin has extruded; a very thin layer of fibrous tissue, parallel to the surface of the wound, connects the two parts, the layers of which, somewhat bent, end in a shrunken intermediate layer. The pigment, that was released from the iris, which is firmly attached to the cornea opposite the scar, has penetrated not only into the original cut surface, but also into the cornea and even into the superficial epithelium.

VI. Experiment. L a r g e g r a y r a b b i t with light blue iris. On November 22nd, a thin piece of mucosa from the inner surface of the lip was brought in and placed with the wounded side downward on the upper quadrant of the iris.

The aqueous fluid has drained away, it turns out, but soon returns. In a short while, with mild inflammatory symptoms, the implanted piece is covered in exudate, through which it shines through as red. When organizing, the exudate contracts and together with the implanted part, forms a small, homogeneous tumor, which is connected to the iris and onto which the iris vessels extend.

A few days later, as the tumor thickens and approaches the cornea, some scleral vessels have grown into the cornea, opposite the location where the tumor is now touching the cornea and acquires a mother-of-pearl sheen.

As the tumor evidently develops further, one sees that some of the threads by which it was connected to the iris are tearing. Meanwhile, the eye shows hardly any signs of irritation; the media are transparent; the fundus is normal. The pupil is narrow and slightly pulled upwards. On April 5, the eye is removed and treated in the regular way.

On sectioning, it is shown that the spherical tumor has caused the cornea to become very thin and has pushed the upper quadrant of the iris deep into the eye, so that the crystalline lens has also become displaced. Everything can now be seen clearly on a transverse cross-section.

The main body of the tumor consists of epithelial cells. At the border at 1 (Fig. 8) are nuclei and small nucleated cells (no layer of cylindrical cells); next (2) are a number of very regular, transparent nucleated cells, which are replaced inwards by very large, flat, almost cornified cells (4), which make up the main mass. The cell mass has shrunken due to Müller's fixative and alcohol and has fallen apart on most sections (1). Anteriorly (at 5), the cells proliferate into the cornea, and Descemet’s membrane is missing (d.). The corneal tissue contains relatively large vessels with very thin walls.

The iris J J' J" forms a very thin layer around the tumor. J is the edge of the pupil, from where we can follow the iris, which covers the tumor at the back up to the vicinity of the cornea (6), where it turns around and continues as the posterior border of the very shallow anterior chamber, towards the area where the Descemet’s membrane splits and connects to the iris as the pectinate ligament. On the inner surface of the iris, the implanted mucosa M is, like the iris, rich in vessels, the structure of which can still be recognized sufficiently, and in which individual thin, extensive, transverse muscle bundles run (at 8 and 8'), which were cut off from the lip together with the mucosa. More anteriorly (at v), the tumor is only surrounded by a thin layer of newly-formed fibrous tissue, which is separated from Descemet’s membrane by a more transparent layer (v). This more transparent layer continues beyond v" on the inner surface of Descemet’s membrane and was probably connected with v'", a similar tissue connected to the iris.

The development of the tumor is clearly visible. The implanted mucosa has adhered to the iris. The epithelium, which was turned forward, found the right conditions for strong proliferation, in which it early on reached the cornea, excavated and thinned
it , and in the face of the resistance encountered, especially the iris (which initially formed cords around the tumor connected to the cornea ), pushed backwards and caused these cords to tear to a large extent, so that on the surface of the tumor facing the pupil only a very thin layer of newly-formed fibrous tissue remained. The newly formed epithelium seems specifically to arise from the opposite side 1 and 2. Meanwhile, young cells are also visible in the cornea (at 5).

For more than one reason, the experiments reported here are incomplete. If we had had enough time, we would have liked to have implanted other tissues into the anterior chamber and particularly tried to implant periosteum and study the effect on the bulbar conjunctiva and the corneal epithelium. Moreover, extrusion of the implanted tissues occurred frequently, thereby preventing us from reaching our goal. And where they did adhere and continued to develop, we would have liked to have examined them at different periods of development and especially continued the observation in living animals for a longer time: in humans, cysts often do not develop until a few years after the injury.

However, our results are important enough.

1. As expected, dead bodies cannot florish. A piece of cork was expelled with perforation of the cornea and iris prolapse. A piece of paper was pushed forward into the corneal tissue and seemed at the brink of perforation. What is remarkable in these cases is the spread of the pigment grains from the iris into the cornea and even into the epithelium covering the cornea. Of this epithelium, sometimes only the deepest layer has retained its position, apparently with peculiar changes in shape (Fig. 3).

However, another option is the development of a capsule around such foreign bodies in the eye chamber. Sometimes this only consists of fibrous tissue. But around a rolled-up paper (Experiment I.) we also saw how thick layers of epithelium developed within the capsule, the final result of which would be of the utmost importance to observe. The origin of this regular epithelium is, as we have noted above, still obscure.
2. Regarding hairs, we know that the anterior chamber of the eye tolerates their presence fairly well. In one of our experiments, however, it was found that a hair in a rabbit can force its way through the cornea almost without causing any irritation.

Particularly remarkable is the growth caused by another hair that had remained on the iris (Experiment II).
3. Of the living tissues that we introduced into the eye, the piece of cornea and of the periost were expelled, and pan-ophthalmia arose in the dog's eye, when human epidermis had been implanted.

In the other experiments, the living parts that had been introduced became really integral parts of the eye, undoubtedly nourished by it and provided with vessels. Only the skin of the ear seems to be an exception here: it was found to be encysted as a dead tissue (Experiment V).

Even with regard to the skin of the white mouse (Experiment IV), growth and further development have not been proven with certainty. This is, however, clearly evident in a small piece of conjunctiva (Experiment III), which, having fused with the cornea and the iris, has assumed the character of fibrous tissue.

Undoubtedly, the most important thing, with regard to the purpose of our investigation, is the further development of the mucous membrane of the lips (Experiment VI). Here, a tumor developed which is completely identical with the follicular tumor described by Rothmund and Buhl (cholesteatoma pearl, epidermidom) and with Monoyer's epithélioma perlé, with this difference that in our case, the cells look like epithelium and not so much like epidermal cells. The manner and conditions of development are also clearly evident here, whereas those observers only had the opportunity to examine the fully developed cells removed from the eye.

In my opinion, there can be no doubt that the proliferation of this tumor would have resulted in the loss of the eye.

Thus, at least the origin of one of the types of pathological products observed in humans after injuries has been brought to light. The living epithelium, with the base on which it rests, is introduced into the eye and can continue to proliferate within it. The question now is (and the result of Experiment I gives rise to this question) whether a similar tumor could not also form around a lifeless foreign body.

4. With regard to the development of cysts, as I have already mentioned, the experiments carried out do not provide sufficient clarification, as the observed cysts cannot be clearly linked to the capsules that develop around foreign bodies.

It seems to us that our experiments are worth continuing in every respect. It is particularly worth considering the consequences of introducing small pieces of benign and malignant
neoplasms immediately after they have been separated surgically from the living body.
